# Polyamine: A Potent Ameliorator for Plant Growth Response and Adaption to Abiotic Stresses Particularly the Ammonium Stress Antagonized by Urea

**DOI:** 10.3389/fpls.2022.783597

**Published:** 2022-03-23

**Authors:** Song Sheng, Changzheng Wu, Yucheng Xiang, Wenxuan Pu, Shuhui Duan, Pingjun Huang, Xiaoyuan Cheng, Yuanyong Gong, Yilong Liang, Laihua Liu

**Affiliations:** ^1^Key Laboratory of Plant-Soil Interaction of MOE, Department of Plant Nutrition, College of Resources and Environmental Sciences, China Agricultural University, Beijing, China; ^2^Tobacco Research Institute of Technology Centre, China Tobacco Hunan Industrial Corporation, Changsha, China; ^3^Hunan Tobacco Science Institute, Changsha, China; ^4^College of Marine Resources and Environment, Hebei Normal University of Science and Technology, Qinhuangdao, China; ^5^College of Biological and Chemical Engineering, Panzhihua University, Panzhihua, China; ^6^Chongqing Key Laboratory of Big Data for Bio Intelligence, Chongqing University of Posts and Telecommunications, Chongqing, China

**Keywords:** polyamine and arginine, abiotic stress, urea signal, ammonium stress, G-protein-coupled receptor, lipid signaling

## Abstract

Polyamine(s) (PA, PAs), a sort of N-containing and polycationic compound synthesized in almost all organisms, has been recently paid considerable attention due to its multifarious actions in the potent modulation of plant growth, development, and response to abiotic/biotic stresses. PAs in cells/tissues occur mainly in free or (non- or) conjugated forms by binding to various molecules including DNA/RNA, proteins, and (membrane-)phospholipids, thus regulating diverse molecular and cellular processes as shown mostly in animals. Although many studies have reported that an increase in internal PA may be beneficial to plant growth under abiotic conditions, leading to a suggestion of improving plant stress adaption by the elevation of endogenous PA *via* supply or molecular engineering of its biosynthesis, such achievements focus mainly on PA homeostasis/metabolism rather than PA-mediated molecular/cellular signaling cascades. In this study, to advance our understanding of PA biological actions important for plant stress acclimation, we gathered some significant research data to succinctly describe and discuss, in general, PA synthesis/catabolism, as well as PA as an internal ameliorator to regulate stress adaptions. Particularly, for the recently uncovered phenomenon of urea-antagonized NH_4_^+^-stress, from a molecular and physiological perspective, we rationally proposed the possibility of the existence of PA-facilitated signal transduction pathways in plant tolerance to NH_4_^+^-stress. This may be a more interesting issue for in-depth understanding of PA-involved growth acclimation to miscellaneous stresses in future studies.

## Introduction

Polyamine(s) [PA(s)], which was discovered first from human semen by van Leeuwenhoek (1678), is the aliphatic nitrogenous base detected ubiquitously in all living kingdoms and exhibits diverse biological activities through interplaying with negatively charged macromolecules, such as DNA/RNA, proteins, and (membrane-)phospholipids (Vuosku et al., 2018). The major PAs in plants comprise diamine putrescine (Put), triamine spermidine (Spd), and tetraamine spermine (Spm); diamine cadaverine (Cad) and Spm-structural isomer thermospermine (T-spm) represent uncommon PAs that occur only in a particular plant species or in a specific development stage when required for response to environmental fluctuations. In cells, PAs are normally present in free soluble or soluble conjugated (e.g., mostly with hydroxycinnamic acids) or covalently bound insoluble forms (Del Duca et al., 2014), with concentrations up to millimolar ranges reported (Pegg and McCann, 1982). The biological necessity or importance of PAs is emphasized in some critical physiological and molecular processes, including the biosynthesis, structural maintenance, stabilization, and activity/function of proteins and nucleic acids (Park, 2006; Liu et al., 2015), and such PA-related pathways have been increasingly described to involve plant effective alleviation of abiotic stresses (Hussain et al., 2019; Jiang et al., 2020). However, more comprehensive characteristics at a molecular level of PAs acting as an efficacious growth regulator capable of initiating downstream signaling cascades to antagonize diverse stresses remain largely uncovered, despite certain findings of PA-related or -coordinated pathways such as PA-H_2_O_2_, -NO, -ethylene, and Glu-Pro-PA-GABA in plant stress responses (Paschalidis et al., 2019; also refer to the “PA generation and catabolism” section briefly). In this short review, to explore and overview the potential role of PAs in improving plant/crop growth adaption and productivity, we focused mainly on PA metabolism/homeostasis, the role of PA as an internal amelioration component to regulate abiotic-stress adaptions in general and to modulate particularly the ammonium stress that can be greatly relieved by external urea at low concentrations, which has been very recently uncovered (Ke et al., 2020; Liu et al., 2021).

## Polyamine Generation and Catabolism

**FIGURE 1:**
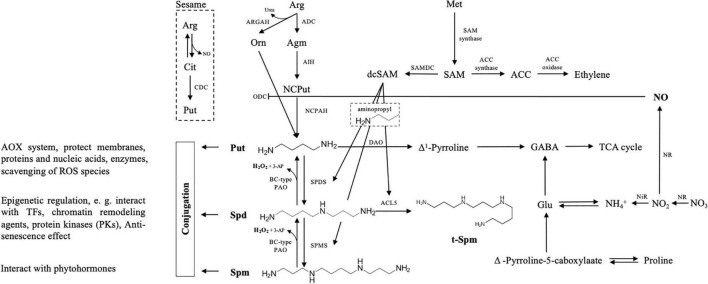
Schematic overview of polyamine (PA) metabolism with interconnection to other metabolic routes. Put biosynthesis may follow three pathways, of which the citrulline (Cit) pathway is reported so far only in sesame plants, where arginine (Arg) is catalyzed by Cit decarboxylase (CDC). The other two main routes include a direct conversion from ornithine (Orn) to Put by the action of ODC, and another indirect pathway with three steps, where ADC, AIH, and NCPAH sequentially decompose Arg, agmatine (Agm), and *N*-carbmoyl-Put, to finally generate Put. In both spermidine (Spd) and spermine (Spm) synthesize pathways, the aminopropyl moieties derived from dcSAM are required, which derived from SAM and catalyzed by the SAMDC enzyme. PA oxidases (PAOs, exhibiting apoplastic activity) catalyze an oxidation-back-reversion reaction (BC-type reaction) to generate H_2_O_2_ that allows “Spm to Spd and to Put” retroconversion. Another catabolite of Put is Δ^1^-Pyrroline triggered by diamine oxidase (DAO). In this pathway, γ-aminobutyric acid (GABA), NO, and proline (Pro) are generated, and NO inhibits ODC activity. Regarding cellular PA-homeostasis, besides free soluble and non-covalently conjugated forms, PAs may also bind to proteins and other molecules to protect the macromolecules, regulate transporter/channel activity and gene expression, or interact with some hormones. This metabolism presentation of PAs in the plant is partially adapted from the studies by Gonzalez et al. (2021) and Spormann et al. (2021).

Biochemically, the synthesis and decomposition of PAs in cells are relatively well characterized [for good reviews, refer to Wang et al. (2019) and Gonzalez et al. (2021)]. The biosynthesis of PAs can be divided into two major steps (Figure 1). In the first phase, three routes for Put production from arginine (Arg) have been revealed in plants: besides the rare citrulline decarboxylase (CDC) pathway described only in sesame plants (Chen et al., 2014), the other two main routes include a direct conversion from ornithine (Orn) to Put by the action of Orn decarboxylase (ODC), and an indirect pathway with three steps, where Arg decarboxylase (ADC), agmatine iminohydrolase (AIH), and *N*-carbamoylputrescine amidohydrolase (NCPAH) sequentially catalyzes Arg, agmatine (Agm), and *N*-carbmoyl-Put, to finally generate Put. Interestingly, the expression of *Arabidopsis ADC2* and *ARGAH* (also known as Arg amidohydrolase) can fully complement the phenotype of Put mutant in yeast, suggesting that arginase exhibits also agmatinase activity besides catalyzing the conversion of Arg (Patel et al., 2017). In general, regarding the physiological roles of ADC and ODC, the ODC pathway seems to be mainly responsible for plant growth, development, organ differentiation, and reproductive stages, while ADC is induced under stress conditions (Fuell et al., 2010).

Once the pathway of Put production is processed, it will be further catalyzed by spermidine synthase (SPDS) to produce Spd and followed by Spm generation by the action of spermine synthase (SPMS). In both Spd as well as Spm and T-Spm synthesis pathways, the aminopropyl moieties derived from decarboxylated *S*-adenosylmethionine (dcSAM) are required (Bagni and Tassoni, 2001; Basu et al., 2014a,b). For dcSAM production, SAM is used by the SAM decarboxylase (SAMDC) enzyme. Simultaneously, SAM also acts as a universal methyl group donor involved in the ethylene metabolic pathway (Harpaz-Saad et al., 2012). On the biological viewpoint, in *Arabidopsis*, auxin regulatory elements are characterized in a promoter region of both *AtSAMDC4* and *AtSAMDC5* genes, leading to a proposal of the functional coordination between PAs, auxin, and ethylene (Majumdar et al., 2017). Besides the “usual” PAs, T-Spm, a tetraamine, and a structural isomer of Spm, produced by thermospermine synthase ACL5, has been found in a wide range of plants from primitive algae to land plants (Harpaz-Saad et al., 2012). The most prominent role of T-Spm characterized so far is its requirement for positively regulating xylem differentiation in vascular plants (Knott et al., 2007; Muniz et al., 2008; Sole-Gil et al., 2019).

The maintenance of PA homeostasis at a proper level is important for/during plant normal growth and development (Rodríguez et al., 2009). To achieve this, processes for the removal of the PA pool and/or its deactivation are necessary. As shown in Figure 1, the catabolism of PAs is mediated by several copper-containing amine oxidases (CuAOs) and flavin-containing PA oxidases (PAOs), contributing to the maintenance of PA internal balance during plant development and stress response (Rodríguez et al., 2009; Jasso-Robles et al., 2019; Yu et al., 2019; Pál et al., 2021). Diamines (e.g., Put and Cad) are mainly catabolized by CuAOs and PAO for Spd as well as for Spm and T-Spm, respectively. In these oxidation pathways, hydrogen peroxide (H_2_O_2_), an oxidation-back-reversion parallel product, may change the homeostasis of internal reactive oxygen species (ROS), thus generating signal(s) involved in regulating plant development and senescence as well as stress response when ROS appears at a proper concentration (Moschou et al., 2012; Wang et al., 2019). In addition to H_2_O_2_, other growth-regulating compounds such as nitric oxide (NO), γ-aminobutyric acid (GABA), and proline (Pro) are also generated in PA catabolism (Figure 1).

Besides the free forms of soluble PAs, covalently conjugated PAs participate in different pathways in plant development (Figure 1). Most importantly, PAs bind to proteins or other molecules due to their cationic nature. PAs, with their non-enzymatic role, protect the macromolecules from the ROS toxicity by participating in a multifaceted antioxidant (AOX) system; in contrast, PAs may act as an epigenetic regulator that alleviates damages from stress or enhance the tolerance by altering the expression of genes involved in the early stress-defensive process (Paul et al., 2021). Although PAs as a nitrogen-containing biochemical agent/intermediate play a critical role in interconnecting different metabolic pathways as stated above, in this study, we would like to especially suggest and discuss a novel finding that PAs may participate in a regulatory mechanism of NH_4_^+^-stress relief by external urea acting as a putative signal molecule, which has been very recently uncovered by Liu et al. (2021).

## Importance of Polyamine(s) as an Amelioration Agent in Growth Adaption and Stress Response in General

The major stress factors inhibiting plant growth and development include salinity, drought, temperature, excessive/inadequate nutrients, and insect/pathogen impairments. To respond to external stimuli, a time-series process of plant and environment interactions has been well-documented. On the perception of the stress information by cells *via* a plasma-membrane locating receptor, signal transduction will be accomplished through the actions of various components, e.g., membrane lipids, second messengers [e.g., Ca^2+^, cyclic adenosine monophosphate (cAMP)], ROS, hormones, protein kinases, and phosphatases, until the activation of a transcription factor required for functional gene expression (Kazemi-Shahandashti and Maali-Amiri, 2018).

Despite being a growth regulator and numerous reports describing PA-affected growth phenotypes under environmental stresses (Supplementary Table 1), comprehensive regulatory mechanisms implicated in PA biosynthesis and catabolism are largely limited. In fact, plants are able to actively respond to inter- and/or intracellular signals through the alteration of PA homeostasis, which is greatly influenced by their intracellular synthesis, catabolism, conjugation, and long-distance translocation in the xylem and phloem, as well as intracellular and organelle exchange of free PAs (Antognoni et al., 1998; Moschou and Roubelakis-Angelakis, 2014; Handa et al., 2018; Nguyen et al., 2018; Podlešáková et al., 2018). PAs are found in all organelles of plant cells but with specific distribution within different cell compartments (reviewed by Kuznetsov et al., 2006). In rice, maize, wheat, and *Arabidopsis*, Spd is mostly accumulated in both leaves and roots, while higher Spm and Put are detected, respectively, in leaves and roots (Pál et al., 2021); in carrot cells, Spm is found to be abundant in the cell wall but Put in the cytoplasm (Cai et al., 2006). Such distribution patterns of PAs may indicate their distinct or specific functions in relation to PA biochemical properties. Being polycationic and ubiquitous aliphatic amines, both Spm and Spd are capable of binding to plasma membrane phosphatidylinositol 4,5-bisphosphate (PIP_2_) (Coburn, 2009), which is a precursor for generating regulatory/signal molecules, i.e., inositol 1,4,5-trisphosphate (IP_3_) and diacylglycerol (DAG), which stimulate multiple protein kinases, transcription, and mRNA processing. Simultaneously, PA production can also be activated by G-proteins and influenced by external cAMP (Choi et al., 1997; Fujiwara et al., 2006), thus triggering phospholipase D (e.g., PLDδ) to generate a signaling molecule phosphatidic acid (Zarza et al., 2019); meanwhile, from PA biosynthesis and catabolism, other signal molecules such as H_2_O_2_, and NO can be synthesized. In a word, the data summarized from such studies indicate the biological significance of PAs that serve as a potent internal mediator/regulator similar to other phytohormones to orchestrate plant growth and development as well as stress responses (Moschou and Roubelakis-Angelakis, 2014; Cai et al., 2015; Liu et al., 2015; Hasan et al., 2021; Spormann et al., 2021). In Supplementary Table 1, we list some nice case studies showing that PAs exert their roles in ameliorating different abiotic stresses.

## Polyamine Acts as a Significant Physiological Component in the Mitigation of Nh_4_^+^-Stress by Urea

Although ammonium represents a principal soil inorganic N-source assimilated by plant cells with a less energy cost compared with that of nitrate (Britto and Kronzucker, 2002; Esteban et al., 2016), and it may even be beneficial to the growth of some NH_4_^+^-preferential/-tolerant species or genotypes under certain environmental stresses such as salinity, drought, and pathogen attack (Marino and Moran, 2019), ammonium toxicity/stress has long been recognized as one of the critical factors inhibiting growth and productivity of many plants/crops, particularly under conditions of using NH_4_^+^- or organic fertilizers or higher NH_4_^+^-containing hydroponic culture for crops/vegetables. To improve plant adaptation to NH_4_^+^ and understand an underlying mechanism(s) of paradoxical effects of higher NH_4_^+^ on plant growth, Liu et al. (2021) has for the first time reported that external urea at low concentrations (<0.1 mM) is sufficient to remarkably attenuate the toxicity of NH_4_^+^ (3 mM) to cotton, with showing a much lower internal Arg than control plants without urea supply. A similar phenomenon is further evidenced in monocot rice and dicot *Arabidopsis* and tobacco (Ke et al., 2020), leading to a suggestion of the existence of a possible genetic/molecular mechanism(s) in higher plants. More interestingly, the growth of cotton roots (also a whole plant) is markedly improved by root urea pretreatment up to 12 h; and split-root experiments reveal a transmittable growth stimulation by urea from one half with continuously low urea supply to another without urea. Such findings assume that external urea is likely to act as a signal substance to specifically elicit signaling for plant detoxification under NH_4_^+^-stress and consequently enhance plant (roots) growth (Liu et al., 2021).

In higher plants, certain genes responsible for PA production are, in fact, found to participate in urea-antagonizing NH_4_^+^-stress, although a precise molecular identity supporting urea-mediated relief from NH_4_^+^-toxicity is experimentally not yet established. As reported by Liu et al. (2021), NH_4_^+^-grown cotton treated with urea shows around 50% reduction in Arg content and 2.5-fold higher PA (Spd + Spm; to be published as a separate article) as compared to those without urea provision; and such measurements are consistent with the observations of a marked transcriptional upregulation of four genes (i.e., *ADC1*, *ADC2*, *SPDS*, and *SPMS*) that are required for Arg decomposition and PA conversion (Liu et al., 2021). Since the expression of three high-affinity NH_4_^+^-transporter homologs (i.e., *AMT1.1/1.2/1.3*) remains generally low with/without urea supply to the ammonium media (Liu et al., 2021), an internal PA elevation level by urea supply in plants should not much affect or repress AMT1s system involved in NH_4_^+^-detoxification, unlike the observation of thymol-ameliorated NH_4_^+^-stress *via* suppressing PA oxidase-derived H_2_O_2_ and modulating AMT1s in rice (Guo et al., 2021). Comparably, in the animal system, an interrelation between urea-cycle activity and PA biosynthesis has been well characterized in controlling certain critical cell processes. For instance, p53 protein, a tumor suppressor, represses the urea cycle by the downregulation of ARG1, carbamoyl phosphate synthetase 1 (CPS1), and Orn transcarbamylase (OTC) to decrease the capability of ammonia elimination, thus inhibiting tumor growth (Li et al., 2019); in parallel, the activity of ODC is suppressed at mRNA level to limit PA generation in human-body tissues (Li et al., 2019). However, there is no evidence *in planta* depicting the effect of the alteration of the expression of PA metabolic-pathway genes on modulating of NH_4_^+^-toxicity, although many studies have shown diverse biological actions of PA in growth/development and stress responses by the misexpression of PA biosynthesis-/catabolism-related molecular components in different species (Alcázar et al., 2010; Moschou et al., 2012). Nevertheless, an alteration of endogenous PA concentrations by external or internal urea (including urea cycle metabolism) may be a crucial physiological player that is required for regulating urea-elicited molecular and cellular processes, including the suppression of NH_4_^+^-stress to the plants by the exogenous urea.

## Possibility of Polyamine-Involved Signaling Process in Plants for Urea-Triggered Relief From Ammonium Stress

At present, despite a lack of direct genetic evidence elucidating the necessity of the role of PA in a mechanism of urea-antagonized NH_4_^+^-toxicity, there are some molecular and physiological cues to support the possibility of PA-integrated putative urea-signaling process. Given a role for urea acting as an external elicitor that might initiate signaling for ameliorating NH_4_^+^-stress, as suggested by the observations from Ke et al. (2020) and Liu et al. (2021), there should be a membrane-spanning receptor for perceiving urea signal. For this hypothesis, the G-protein-coupled receptor (GPCR) would be one of the candidates. In humans, adenosine being a tissue-protective molecule plays a role in regulating various biological processes particularly in nervous and cardiovascular systems, and most of the protective functions of adenosine are ascribed to its interaction with the adenosine A1 receptor, which is one of the four GPCR homologs that are recognized by external adenosine (May et al., 2005). Using a method of uncoupling membrane receptors from G-proteins for ligand screening, May et al. (2005) also reported that urea (at 100 μM) could abolish or increase the binding of an agonist (adenine) or antagonist [8-cyclopentyl-1,3-dipropylxanthine[dipropyl-2,3] (DPCPX)] to the A1 receptor. This argues that urea may itself modify the ligand-binding property and/or the function of the A1 receptor or that urea might target to or be recognized by such GPCR proteins, thus initiating downstream signaling cascades specific for a biological response.

**FIGURE 2:**
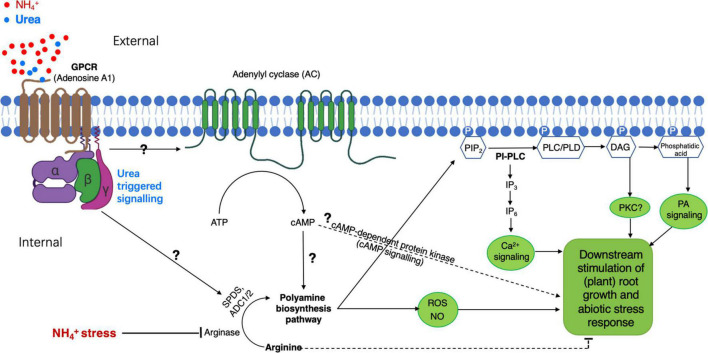
A proposed model of PA-participated signaling pathway in plants for urea-elicited alleviation of ammonium stress. Heterotrimeric guanine nucleotide-binding proteins (G-protein) anchor to the inner leaflet of the plasma membrane and are coupled to a putative G-protein-coupled receptor (GPCR) specific for external urea. The GPCR recognizes and binds to urea, leading to its conformation change for the activation of the G-protein(s), by which a membrane-bound adenylyl cyclase (AC) is activated to generate a second messenger cAMP. Increased cAMP activates downstream some unknown molecular component(s) to eventually modify biosynthesis pathways for PA production; the increased cAMP might also activate cAMP-dependent protein kinase to directly modulate the stress response. The subsequent activation of phospholipase C or D (PLC or PLD) by PAs promotes the generation of PIP2-derived signal molecule IP3/DAG and/or phosphatidic acid in the cytosol, resulting in the activation of an unknown inner membrane ion-channel(s) for Ca^2+^ release. An increase in cytosolic Ca^2+^ will initiate different cellular processes for specifically regulating plant growth and development under given conditions, e.g., NH_4_^+^-stress with the presence of urea. This model postulated for PA-involved urea-antagonized NH_4_^+^-stress is based on our recent findings (Ke et al., 2020; Liu et al., 2021) and intensive surveys of related publications cited in this study. PIP2, phosphatidylinositol 4,5-bisphosphate; IP3, inositol-triphosphate; DAG, diacylglycerol.

In plants, the proteomic profiling with cultured rice cells overexpressing *OsRac1*, a member of GTPase proteins that serve as a molecular switch to activate heterotrimeric G-protein α-subunit (G_α_) in defense response (Suharsono et al., 2002), has revealed a remarkable induction of PA biosynthesis-responsible enzymes, e.g., arginase and Spd synthesis 1 (Fujiwara et al., 2006). Because G-proteins and GPCR are widely conserved in living systems including the plants (Hooley, 1999; Pandey and Assmann, 2004; Taddese et al., 2013), their sophisticated functions in regulating plant growth and development responsive to external challenges such as NH_4_^+^-stress remain largely unknown. Thus, based on our current understanding, we would hypothesize (Figure 2) that a GPCR(s) might be a potential candidate acting as a “urea receptor or sensor”-mediating external urea-elicited signal transduction route to finally suppress NH_4_^+^-stress, in which PA is required as one of the key components as supported by the observations from Fujiwara et al. (2006) and Liu et al. (2021); However, how external urea (or its elicited signaling) upregulates PA-producing enzymes at a (post)transcriptional and/or (post)translational level remains to be explored.

As suggested above, the elevation of internal PA level appears to be an outcome of the early response of urea-antagonized NH_4_^+^-stress. To our knowledge, some valuable hints allow us to further appreciate PA-related downstream physiological and molecular players. Studies with renal cells have demonstrated that the urea-inducible expression of *Egr-1*, which is one of the immediate-early genes coding for a zinc-finger transcription factor, is an outcome of phosphoinositide phospholipase C (PI-PLC)-related signaling, which is implicated in the sequential activation of the receptor tyrosine kinase (RTK)-specific PLC-γ, the release of a second messenger inositol-triphosphate (IP_3_, triggering intracellular Ca^2+^ signal delivery), and the activation of protein kinase C (PKC). This leads to a proposal of the possibility of a cytosolic and/or cell surface urea-sensing RTK (Cohen et al., 1996; Zhao et al., 2003). In higher plants, despite no counterpart identified so far for IP_3_-receptor and PKC(s), some important homologous components (e.g., the activity of PLCs, receptor-like tyrosine kinases, and Ca^2+^ channels linking with lipid catabolites including IP_3_) in the PI-PLC or lipid-signaling pathway(s) have been identified in many species, and the activation of PI-PLC signaling events by external stimuli is shown to direct various biological processes including plant stress responses (Echevarría-Machado et al., 2004; Vossen et al., 2010; Munnik and Nielsen, 2011). Interestingly, the activity of PLC in the roots of *Catharanthus roseus* and *Coffea arabica* is revealed to be markedly and differently influenced by PAs (Echevarría-Machado et al., 2004). Besides a relationship between PLC and PA, a strong PA dosage-dependent inhibition of the PLD action in tobacco is observed by the assay of PLD activity with the presence of G-proteins (Lein and Saalbach, 2001); in *Arabidopsis*, extracellular Spm can trigger a rapid phosphatidic acid response through PLDδ activation (Zarza et al., 2019).

Furthermore, studies with human cell lines have provided evidence that the inner membrane leaflet PIP_2_ of the plasma membrane is rapidly hydrolyzed by PLC and/or PI(4,5)P_2_ 5-phosphatase to form the signal molecule IP_3_ and DAG, particularly in the G-protein-linked receptor-activated cells (Baron et al., 1989; Coburn, 2009). In addition, the finding of PA (Spm and Spd) binding to PIP_2_ and also to polybasic membrane(-anchoring) proteins using electrostatic interaction (Baron et al., 1989; Coburn, 2009) further emphasizes the critical functions of PAs in regulating molecular and cellular processes *via* the alteration of various PIP_2_-dependent signaling and channels/transporters (e.g., K^+^ and Ca^2+^) (refer to a review by Coburn, 2009). Nonetheless, based on the current research data, there would be a biological connection between PA action and PI-/PIP_2_-PLC/PLD lipid signaling. Thus, a preliminary model of “urea-GPCR-PA-PLC/PLD-IP_3_/DAG…” signal transduction pathway could be proposed in the plant for urea-attenuated NH_4_^+^-stress (Figure 2). However, if this model would hold true, how external urea eventually improves the growth of the NH_4_^+^-stressed plants at cellular/tissue levels needs to be largely investigated.

## Discussion and Future Perspectives

Abiotic stresses from environments have been estimated to result in an average yield reduction by over 50% for most major crops (Alcázar et al., 2010), prompting researchers to intensively explore the mechanisms underlying plant stress response. In recent years, PA has been paid more attention due to our increasing understanding of its important and multifarious roles in the regulation of plant growth, development, and tolerance to major abiotic/biotic impairments. However, compared with studies in the animal system, where diverse functions of PAs have been profoundly appreciated at physiological, molecular, and cellular levels, which are implicated in cell motility, actin-cytoskeleton reorganization, membrane trafficking, Ca^2+^ signaling, phosphatases, multiple kinases in various signal transduction cascades, and transporters/ion channels (Coburn, 2009), most investigations with plants of the PA effect on growth adaption/withstanding to, e.g., abiotic stresses (including heat, drought, cold, salt, and minerals) were conducted mainly at physiological aspects in relation to PA metabolism, homeostasis, and its metabolic downstream products such as ROS. The elevation of an internal PA concentration seems to be a common physiological marker for plant stress response and can improve the growth, providing probably a useful approach to enhance crop growth and productivity under a given stress condition by external PA supply or more internal synthesis of PA through genetic manipulation. More noticeably, besides the ability of PA to directly bind to macromolecules (e.g., protein, DNA, and RNA) to regulate cellular processes (Cai et al., 2015), being a growth regulator or phytohormone-like compound, PA in plant cells is strongly suggested to occupy an essential position in different signal transduction pathways, similar to those found in animal systems. From a molecular physiology point of view, this may be a more important research issue for the in-depth understanding of PA-involved growth acclimation to various stresses in future studies, for instance, in the case of urea-attenuated ammonium stress, namely, how PA exerts its potent action in the process of NH_4_^+^-detoxification by urea signaling as proposed in Figure 2 is a great interest.

## Author Contributions

LL, SS, CW, YX, WP, and SD provided an outline, prepared the manuscript, and produced the figures and tables. PH, XC, YG, and YL provided an outline, screened the literature, and guided the discussion. All authors have read and agreed to the published version of the manuscript.

## Conflict of Interest

WP and PH were employed by company China Tobacco Hunan Industrial Corporation. The remaining authors declare that the research was conducted in the absence of any commercial or financial relationships that could be construed as a potential conflict of interest.

## Publisher’s Note

All claims expressed in this article are solely those of the authors and do not necessarily represent those of their affiliated organizations, or those of the publisher, the editors and the reviewers. Any product that may be evaluated in this article, or claim that may be made by its manufacturer, is not guaranteed or endorsed by the publisher.
