# Supplementary material for: Polyamine: A Potent Ameliorator for Plant Growth Response and Adaption to Abiotic Stresses Particularly the Ammonium Stress Antagonized by Urea
Source: Front Plant Sci. 2022 Mar 23;13:783597. doi: 10.3389/fpls.2022.783597 (PMC8988247; doi:10.3389/fpls.2022.783597)
Supplement: Supplementary file 1 [file Table_1.docx]

| **Substance application** | **Applied concentration** | **Affected gene/pathway or protein/enzyme activity** | **Species** | **Reference** | **Action or mitigation effect** |
| --- | --- | --- | --- | --- | --- |
| Urea | 50 µM | ADC1/2, SPMS, SPDMS(up) | Cotton, | Liu et al., 2021 | NH_4_^+^ toxicity |
| Urea | 50–100 µM | N/A | Arabidopsis, Tobacco, Rice | Ke et al., 2020 | NH_4_^+^ toxicity |
| Spd | 0.5 mM | ADC | Rice | Jiang et al., 2020 | Aluminum toxicity |
| Spd | 0.1 mM | ROS scavenging enzymes | Cucumber | Kubiś, 2007 | Water stress |
| Put | 1 mM | ADC1/2 | Tomato | Ding et al., 2021 | Cold stress |
| Silicon | Sodium silicate fertigation  250 mL per pot (1.67 mM) | PAO, DAO (down) | Cucumber | Szegó et al., 2021 | Oxidative stress |
| Put orSpd | 0.75 mM | N/A | Beet, Tegetes | Bais et al., 2000 | Growth & metabolic change |
| Spd | 1 mM | N/A | Rice | Saleethong et al., 2013 | Salt stress |
| Spd | N/A | N/A | *Citrus sinensis* | Saleem et al., 2008 | Enhance growth |
| PAs | N/A | N/A | *Nepeta cataria* | Yang et al., 2010 | Enhance growth |
| Spd or Spm | 0.5 mM | SOD, CAT | *Rosa deamascena* | Hassan et al., 2018 | Drought stress |
| Spd | 1 mM | SOD, CAT, POD | *Calendula officinalis* | Baniasadi et al., 2018 | Salt stress |
| Spd or Spm | 1 mM | SOD, CAT, POD | Wheat | Guo et al., 2020 | Heat stress |
| PAs |  |  |  | Hasanuzzaman et al., 2019 | Metal stress |
| Spm | 0.01–5 mM | N/A | In 20 species | Reviewed by Hasan et al., 2021 | Drought stress |
| Put | 1 mM | ODC | Strawberry | Tarenghi and Martin-Tanguy, 1995 | Floral development |
| Zeatin | 20 µM | ADC, SPDS, SAMDC, DHS (Deoxyhypusine synthase) | Wheat | Alharby et al., 2020 | Salinity and drought stress |
| Spm | 100 ppm | N/A | Canola | Orabi et al., 2020 | Salinity stress |
| Put/Spd/Spm | 10–1000µM | ROS | *Pyrus communis* | Aloisi et al., 2015 | Pollen tube elongation |

**Supplementary Table 1.**PA directly or indirectly mediates a tolerance-enhancement in different plant species

**References cited only in the Supplementary Material**

Alharby, H. F,Alzahrani, Y. M., Rady, M. M. (2020). Seeds pretreatment with zeatins or improved hormonal contents, polyamine gene expression, and salinity and drought tolerance of wheat. *Int. J. Agri. Biol.24*, 714–724. doi: 10.17957/IJAB/15.1491

Aloisi, I., Cai, G., Tumiatti, V., Minarini, A., Del Duca, S. (2015). Natural polyamines and synthetic analogues modify the growth and the morphology of *Pyrus communis* pollen tubes affecting ROS levels and causing cell death. *Plant Sci.* 1–28.doi: 10.1016/j.plantsci.2015.07.008

Bais, H. P., Madhusudhan, R., Bhagyalakshmi, N., Rajasekaran, T., Ramesh, B. S., and Ravishankara, G. A. (2000). Influence of polyamines on growth and formation of secondary metabolites in hairy root cultures of *Beta vulgaris* and *Tagetes patula*.*Acta Physiol. Plant* 22, 151–158. doi:10.1007/s11738-000-0070-x

Baniasadi, F., Saffari, V. R., Moud, A. A. M. (2018). Physiological and growth responses of *Calendula officinalis* L. plants to the interaction effects of polyamines and salt stress. *Sci. Hort.234*, 312–317. doi: 10.1016/j.scienta.2018.02.069

Ding, F., Wang. C., Xu. N., Wang, M.-L., Zhang. S.-X. (2021). Jasmonic acid-regulated putrescine biosynthesis attenuates cold-induced oxidative stress in tomato plants. *Sci. Hort*. 288, 110373. doi.org/10.1016/j.scienta.2021.110373

Hassan, F. A. S., Ali, E. F.,Alamer, K. H. (2018). Exogenous application of polyamines alleviates water stress induced oxidative stress of *Rosa damascena Millervar. trigintipetalaDieck*. *South African J. Bot.116*, 1–7. doi: 10.1016/j.sajb.2018.02.399

Hasan, M. M., Skalicky, M., Jahan, M. S., Hossain, M. N., Anwar, Z., Nie, Z. F., Alabdallah, N. M., Brestic, M. Hejnak, V., Fang, X. W. (2021). Spermine: its emerging role in regulating drought stress responses in plants. *Cells10*, 1–15. doi.org/10.3390/cells1002026

Hasanuzzaman, M., Alhaithloul, H. A. S., Parvin, K., Bhuyan, M. H. M. B., Tanveer, M., Mohsin, S. M., Nahar, K., Soliman, M. H., Mahmud, J. A., Fujita, M. (2019). Polyamine action under metal/metalloid stress: regulation of biosynthesis, metabolism, and molecular interactions. *Int. J. Mol. Sci.20*, 3215. doi: 10.3390/ijms20133215

Jing, J.-G., Guo, S.-Y., Li, Y.-F., Li, W.-H. (2020). The alleviating effect of exogenous polyamines on heat stress susceptibility of different heat resistant wheat (*Triticum aestivum* L.) varieties. *Sci. Rep.* 10: 7467. doi.org/10.1038/s41598-020-64468-5

Kubiś, J. (2007). Exogenous spermidine differentially alters activities of some scavenging system enzymes, H. *J. Plant Physiol.* 165, 397–406. doi:10.1016/j.jplph.2007.02.005

Orabi, S. A., El Shahawy, T. A., Sharara, F. A. (2020). The polyamine spermine in retarding salinity induced stress in canola. *Middle East J. Applied Sci.10*, 1–15. doi: 10.36632/mejas/2020.10.1.12

Saleem, B. A., Malik, A. U., Anwar, R. (2008). Exogenous application of polyamines improves fruit set, yield and quality of sweet oranges. *Acta Hort.774*, 187–194. doi: 10.17660/ActaHortic.2008.774.23

Saleethong, P., Sanitchon, J., Kong-ngern, K., Theerakulpisut, P. (2013). Effects of exogenous spermidine (Spd) on yield, yield related parameters and mineral composition of rice (*Oryza sativa* L. *ssp. indica*) grains under salt stress. *Australian J. Crop Sci.7*, 1293–1301. doi=10.1.1.1089.4261&rep=rep1&type=pdf

Szegõ, A., Mirmazloum, I., Pónya, Z., Bat-Erdene, O., Omran, M., Kiss-Bába, E., et al. (2021). Downregulation of polyamine and diamine oxidases in silicon-treated cucumber. *Plants* 10, 1–11. doi:10.3390/plants10061248

Tarenghi, E.; Martin Tanguy, J. (1995) Polyamines, floral induction and floral development of strawberry (*Fragaria ananassaDuch.*). *J. Plant Growth Regul.17*, 157–165. doi: 10.1007/BF00024176

Yang, Y. K., Lee, S. Y., Park, W. T., Park, N., Park, S. U. (2010) Exogenous auxins and polyamines enhance growth and rosmarinic acid production in hairy root cultures of “*Nepeta cataria*” L.*Plant OmicsJ*ournal,3 (6): 190–193
